# Predictors of Healthcare-Associated Bloodstream Infections in Subjects Hospitalised from the Emergency Department for Non-Infectious Disease

**DOI:** 10.3390/jcm15124771

**Published:** 2026-06-19

**Authors:** Andrea Fabbri, Ayca Begum Tascioglu, Flavio Bertini, Barbara Benazzi, Roberto Martello, Danilo Montesi

**Affiliations:** 1 Emergency Department, Local Health Agency of Romagna, Presidio Ospedaliero Morgagni-Pierantoni, Via C Forlanini 34, 47121 Forlì, Italy; bbenazzi1@gmail.com; 2 Department of Computer Science and Engineering, University of Bologna, Mura Anteo Zamboni 7, 40126 Bologna, Italy; aycabegum.tascioglu2@unibo.it (A.B.T.); danilo.montesi@unibo.it (D.M.); 3 Department of Mathematical, Physical and Computer Sciences, University of Parma, Parco Area delle Scienze 53/A, 43124 Parma, Italy; flavio.bertini@unipr.it; 4 Health Directorate of Local Health Agency of Romagna, Presidio Ospedaliero Morgagni-Pierantoni, Via C Forlanini 34, 47121 Forlì, Italy; roberto.martello@auslromagna.it

**Keywords:** key features, emergency department, mortality, nosocomial infections, hospitalisation

## Abstract

**Background**: Healthcare-associated bloodstream infections (HABSIs) are among the main categories of nosocomial infections. This analysis aims to identify the clinical characteristics of patients in the emergency department (ED) who will develop a HABSI during their hospital stay. **Methods**: Main outcome measures were HABSI and the cumulative survival rate at 30 days. The features tested in a logistic model were age, sex, vitals by the National Early Warning Score (NEWS), priority levels, main complaints, comorbidities by the Charlson Comorbidity Index (CCI), trauma-related disease, main diagnosis and ED length of stay. **Results**: In 414 (2.3%) out of 18,304 patients, aged 75 (16) years, mean (SD), a diagnosis of HABSI was recorded. HABSIs occurred in subjects with main diagnosis of diseases of the respiratory system (N = 116; 28.0%), digestive system (N = 72; 17.4%), and circulatory system (N = 68; 16.4%). The main key clinical features selected by the logistic model were: NEWS > 6, diagnosis of neoplasms, CCI > 4, and diagnosis of diseases of the digestive system. The ROC curve for the HABSI risk score was 0.703 ± 0.027 in predicting the outcome, (sensitivity 79%, specificity 51%, at optimal cut-off score). The overall hazard mortality risk was twofold higher in patients with HABSIs (hazard ratio: 2.319; 95% confidence interval: 1.871–2.875; *p*-value: <0.001). The overall 30-day survival rate was lower among patients with HABSIs (33%) vs. non-HABSI patients (62%). **Conclusions**: A group of main clinical features in subjects without suspect of infectious disease in the ED are associated with HABSIs. These features negatively impact survival rate during hospital stays.

## 1. Introduction

### 1.1. Background/Rationale

Healthcare associated infections (HAIs) represent a major public health problem globally [1]. Annual surveillance reports from the European Centre for Disease Prevention and Control (ECDC) [2] indicate that the most common HAIs are pneumonia, followed by urinary tract infections, surgical site infections, bloodstream infections, gastrointestinal infections, and skin and soft tissue infections [2].

Healthcare-associated bloodstream infections (HABSIs), even though they account for only a fraction of these (11.9%), appear to have a very unfavourable outcome, as they can lead to sepsis, particularly in critical cases [2,3]. Blood cultures are the gold standard for diagnosis, but there is still no conclusive evidence regarding the optimal timing for their collection [4].

Early identification of high-risk subjects in the ED could lead to earlier diagnosis and treatment, thus reducing the impact of these complications and unfavourable outcomes [2,5]. In patients presenting to the ED with suspected infectious disease, several key predictive factors—namely, cases involving respiratory failure, the use of vasopressors, neutrophilia, thrombocytopenia, indwelling venous catheters, fever, urinary tract infection or endocarditis—have already been found to be associated with positive bacteraemia of any origin [6].

### 1.2. Objectives

The present study aimed to explore in depth the main clinical characteristics of subjects admitted to an acute care hospital from the ED with non-infectious disease, who developed a HABSI.

## 2. Materials and Methods

### 2.1. Study Design, Setting

This is a retrospective multicentre analysis of all digital health records collected from the official registry from seven 1st-level EDs of the Local Health Agency of Romagna, Italy, between 1 June 2023 and 31 December 2024 (18 consecutive months) including 495,631 encounters/1,172,853 inhabitants.

This study was approved by the Local Ethics Committee (CEROM) and conducted in accordance with the ethical standards outlined in the 1964 Declaration of Helsinki and its subsequent amendments [7]. Because patient data were anonymised, informed consent was obtained by the ethics committee. The study was classified as type 4 according to the TRIPOD (Transparent Reporting of a Multivariable Prediction Model for Individual Prognosis or Diagnosis) guidelines [8] and conducted in accordance with them [9].

### 2.2. Patients

To define the target cohort, among 495,631 initial encounters from the electronic health records of the 7 centres, we excluded the following subjects: (a) being discharged home after the ED visit (N = 433,592), (b) having incomplete information (N = 25,635), (c) being admitted to an external hospital (N = 5652), (d) being under 18 years of age (N = 2645), and (e) having died in ED before hospital admission (N = 591).

**Figure 1:**
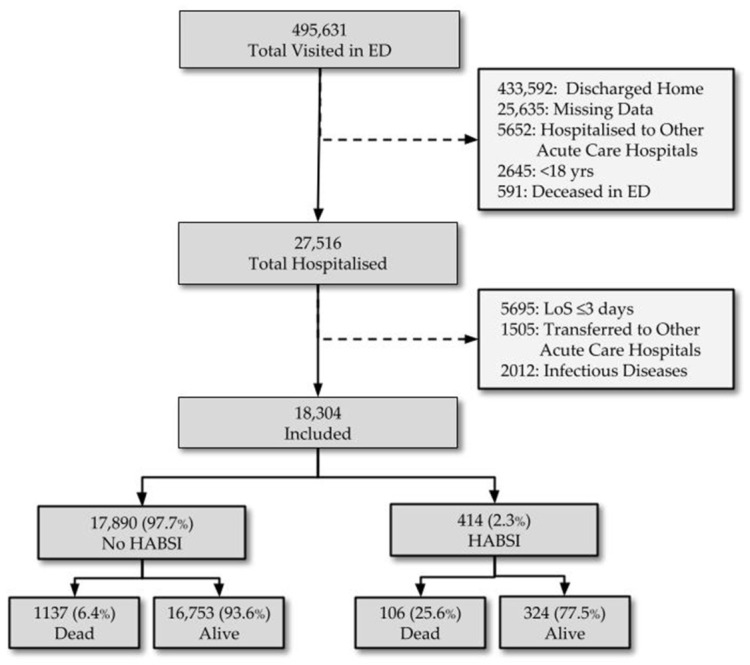
Flow diagram of the study.

Following the hospitalisation, 27,516 subjects were further excluded because: (f) hospital stay ≤3 days (N = 5695), (g) transfer to other hospitals (N = 1505), and (h) first or second diagnosis of a HABSI and primary diagnosis of infectious disease (N = 2012), as ICD9 CM infectious and parasitic diseases diagnosis codes 001 – 139 and those which included diseases generally recognized as communicable, transmissible, or caused by biological agents, spanning intestinal bacterial, viral, fungal and parasitic infections (Figure 1). After excluding all these cases, statistical analysis was performed on 18,304 health records of subjects hospitalised for estimated non-infectious diseases.

### 2.3. Variables

Investigators blinded to patient outcomes extracted the following data from electronic records: age, sex, priority levels and vital signs at arrival in the ED. Vital signs, such as systolic blood pressure, heart rate, respiratory rate and temperature, were considered to calculate the National Early Warning Score (NEWS), which is considered a categorical variable (0–4: low risk; 5–6: medium risk; >6: high risk) [10].

The chief complaints at arrival in ED were assigned using the Canadian Emergency Department Information System (CEDIS) Complaints List (V2.0) classification [11]. All presenting complaints were further divided into two categories, traumatic vs. non-traumatic origin.

The CCI [12] was calculated using information from the free-text fields of the digital health records. It was considered as follows (mild, 1–2; moderate, 3–4; severe, >4), using recently validated selection criteria and disease categories. Age adjustments were applied, with 1 point added for each decade over 40 years of age (e.g., 50–59 years, +1; 60–69 years, +2; 70–79 years, +3, etc.), with these “age points” added to the total CCI score [12].

The ECDC disease categories [2] considered for HABSIs were identified using the following ICD9-CM diagnosis codes: 995.91 [septicemia], 995.92 [sepsis]; 038, 038.4 [septicemia, including specific organ involvement]; 038.8, 038.9, 790.7 [unspecified septicemia/bacteremia]; 038.0–038.1 [streptococcal septicemia]; 038.2 [Gram-negative septicemia].

Subjects with a diagnosis of HABSI were identified as those who had: (a) at least one of the ICD9-CM diagnosis codes included in the ECDC disease category of bloodstream infection [2], (b): hospital stay >3 days [2], (c) subjects with 1st or 2nd diagnosis of non-infectious disease, (d) diagnosis of HABSI during hospital stay only as the 3rd–6th diagnosis, and (e) confirmatory information from clinical, laboratory (first positive blood culture) [13], microbiological and radiological electronic health records.

If a patient had more than one HABSI, it was counted only once. In the statistical model, the following variables were also tested: ED length of stay (in hours), overnight stay in ED between midnight and 8 a.m., and weekend vs. workday.

### 2.4. Statistical Analysis

The data were summarised as counts and percentages. Continuous variables were reported as either the mean (standard deviation, SD) or median [interquartile range, IQR]. Differences in patient characteristics, along with the corresponding 95% confidence intervals (CIs), were calculated using the Agresti–Caffo method [14]. For all statistical analyses, we set the significance level at *p* < 0.001.

The primary outcome measure was HABSI occurrence during hospital stay. The demographic characteristics taken into consideration were age, sex, and comorbidities.

Comorbidities were considered both as individual variables and as a categorised value of CCI > 4. Additional variables tested were NEWS, priority levels (0–4) [15], ICD9-CM main diagnosis codes, trauma and non-trauma related visits, ED length of stay, calculated as the difference between entry and exit times as a categorical value (<6, 7–12, 13–24, >24 h), in-hospital length of stay (iHLoS) (in days), overnight stay (from midnight to 08:00 a.m.), and weekend vs. weekday.

A multivariable logistic model was used for estimating the association between selected variables and HABSIs. The odds ratio (OR) and 95% confidence intervals (95% CIs) were calculated. A risk score was calculated for each patient based on the coefficients computed by the logistic model derived from variables entering the stepwise procedure.

The accuracy of such a risk score was evaluated by the area under the receiver operating characteristic (ROC) curve. The optimal cut-off point score was calculated by the Youden index [16]. To avoid ambiguity, no synthetic data were generated to address missing data; hence, the analysis utilised only complete cases. For feature selection, stepwise feature selection is used [17].

Cumulative survival rate at 30 days for the HABSI vs. non-HABSI category was calculated by the log-rank test (Mantel–Cox test) to compare the survival distributions (%) between the different groups.

Using a two-tailed binomial test, we estimated that, for a study with adequate statistical power and an acceptable margin of error of ±1%, assuming a significance level (α) of 5%, a power of 80%, and an expected prevalence of HABSI cases of 7.7%, as reported in the ECDC’s last publication [2], the required initial sample size would be at least 2731 patients, a lower number of cases than what was considered. The logistic model used a rule of thumb of at least 10 events per variable. A model with 10–20 predictors would require at least 200 cases of HABSI. Assuming an incidence rate of 7.7%, this would be about 2600 patients, in line with the previous estimate. Statistical analyses were conducted in Python (version 3.10.12), with the libraries lifelines (version 0.30.0), sklearn (version 1.6.1) and SciPy (version 1.16.3).

## 3. Results

### 3.1. Study Subjects

The analysis was conducted on 18,304 patients (mean age: 75 (16) years; mean (SD)) hospitalised for at least 3 days with a diagnosis of non-infectious disease. Overall mortality was recorded in 25.6% of subjects who developed avHABSI and in 6.4% of subjects without a HABSI (Figure 1).

Patient characteristics are reported in Table 1, comparing the characteristics of the HABSI group (414 cases; 2.3%) with the non-HABSI group (17,890 cases; 97.7%). Patients with a HABSI were more frequently male, with an even higher prevalence in older age groups (Table 1).

In the HABSI group, the most common diagnoses were: severe sepsis with organ dysfunction (995.92), N = 106 (25.6%); *Escherichia coli* septicaemia (038.42), N = 65 (15.7%), unspecified bacteraemia (790.7), N = 62 (15.0%), and staphylococcal septicaemia (038.19), N = 46 (11.1%).

Similarly, subjects in the HABSI group had a higher proportion of cases with a NEWS score of moderate (4–6) or high risk (>6), as well as a higher proportion of cases with a severe comorbidity index (CCI > 4) (Table 1). The mean EDLoS was 13 (16) hours (mean (standard deviation)), with no difference between the groups, whilst the mean iHLoS was 11 (8) days (mean (standard deviation)), with an average increase of 8 h in cases with a HABSI.

The main diagnoses of non-infectious diseases (Table 2) were, in order of frequency, diseases of the respiratory system (26.0%), diseases of the circulatory system (22.3%), diseases of the digestive system (12.9%), injury and poisoning (11.4%) and diseases of the genitourinary system (7.7%). Diseases of the digestive and genitourinary systems, as well as neoplasms, were more prevalent in the HABSI group than in the non-HABSI group (Table 2).

### 3.2. Predictive Model

**Figure 2:**
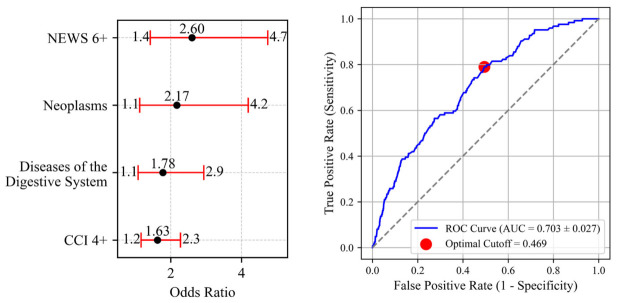
Forest plot (**left**) of the selected variables entered into the multivariable logistic model (**right**) in the association to a HABSI. Data are reported in order of importance as odds ratios and 95% confidence intervals. The grey dashed line indicates the random chance of the area under the curve (AUC) 0.5.

The list of independent variables selected from the logistic model to predict occurrence of HABSI is shown in order of importance in Figure 2.

In order of importance, they were: NEWS > 6, diagnosis of neoplasm, diseases of the digestive system, and a CCI > 4. Among subjects with a primary diagnosis of neoplasm, the most common diagnoses were pancreatic cancer (N = 53; 5.2%), lung cancer (N = 46; 4.5%), and ascending colon cancer (N = 44; 4.3%). Regarding diagnoses of diseases of the digestive system, the most common were acute pancreatitis (N = 193; 8.2%), intestinal obstruction N = 143; 6.1%), and colonic diverticulitis (N = 135 cases, 5.7%).

The ROC curve for the HABSI risk score, calculated using the coefficients from the logistic regression, is shown in Figure 2. The model achieved an accuracy of 0.703 ± 0.027 in predicting the outcome, with a sensitivity of 79% and a specificity of 51%, at the optimal cut-off point of 0.469. This point of the curve was selected to achieve maximum sensitivity and specificity simultaneously.

**Figure 3:**
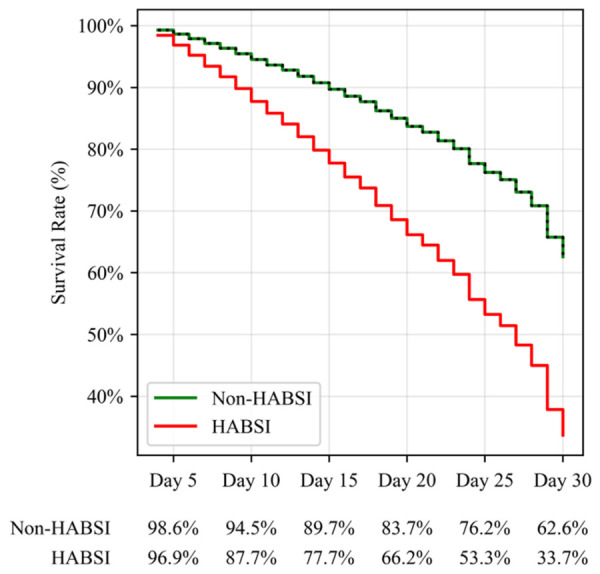
Cumulative survival rate for the two different groups: HABSI (red line) vs. non-HABSI category (green line). The log-rank test (Mantel–Cox test) was used to compare the survival distributions (%) between the different groups.

The 30-day overall survival rate in the Mantel–Cox hazard model risk was significantly reduced in the HABSI (33.7%) group compared to the non-HABSI group (62.6%) (Figure 3), with an increased risk for subjects who developed a HABSI (hazard ratio 2.319; 95% CI: 1.871–2.875; *p*-value: <0.001).

## 4. Discussion

### 4.1. Key Results

The present study shows that some characteristics identified at admission to the ED predict, with a just acceptable level of accuracy, the development of a HABSI during hospitalisation, a serious complication with an increased risk of death.

The variables associated with the development of a HABSI selected by the model, with only moderate overall discriminatory power, tend to favour sensitivity over specificity, because the optimal point of the curve falls at a sensitivity level of 79%, at a specificity of 51%. In other words, the variables support the models’ bias toward sensitivity.

The study protocol considered the group of subjects with a HABSI, selecting them based on criteria defined by the ECDC [2], with the effort to contain false positives, i.e., cases with an infectious disease already present at the time of admission, but with symptoms not yet evident at the time of admission in the ED [18]. The proportion of such cases can, in fact, vary considerably and depends on many factors, given that the latest ECDC report estimates that these cases range from 15.6% (Czech Republic) to 41.2% (Sweden) [2].

In a recent study on over 8000 patients admitted to the ED with a diagnosis of infectious disease, 9.1% of cases had blood culture tests that were positive. The main clinical characteristics included age > 55 years, moderate to severe chronic kidney disease, solid organ cancer, liver disease, a history of chills, and a body temperature above 38.3 °C [7].

We believe that the selection criteria for the cases to be studied (i.e., cases involving a hospital stay of >3 days, a primary diagnosis of a non-infectious disease, a HABSI diagnosis as the third to sixth diagnosis, do not introduce selection bias and minimise the risk of false positives [19]. The inclusion of patients with a hospital stays of >3 days is consistent with the aim of reducing the risk of including infections already present or incubating at admission. Obviously, patients who died within three days of septic shock were also excluded. It is unlikely that these were cases with a primary diagnosis of a non-infectious disease who died from a HABSI complication within ≤3 days.

It should also be emphasised that defining reliable criteria for determining the exact date of HABSI occurrence would, in any case, be highly complex, owing to the many variables that must be considered and the unreliability of diagnosis dates. In selecting cases, we used ICD9-CM coding to identify those falling within the subcategory of healthcare-associated infections.

Although coded administrative data are generally considered inaccurate and unreliable for many HAI outcomes [19], it has proven to have good accuracy in our hands, with a specificity of at least 93%. Although ICD-9-CM codes are administrative by nature, in our series, each HABSI code was individually verified against the medical record by clinicians and administrative staff, by granting consent for the recording. In addition, considering an internal validation, differences in regional health-care organization and coding practices could affect an external validation.

In our dataset, HAIs were identified in 2556 cases (8.9% of all hospitalised cases), a figure that would appear to be in line with the official ECDC registers, which attribute to Italian hospitals a percentage of cases ranging from 7% to 10% of total cases, and of these, a proportion (approximately 18%) with HABSIs [20]. It should also be noted that, due to the incomplete nature of the available information and sometimes regulatory or technical constraints imposed by other institutions, retrospective studies must unfortunately accept a minimal risk of selection bias. Conversely, the application of a multivariate regression model is intended to minimise errors introduced by confounding variables [21].

The NEWS is a leading indicator of 30-day mortality in patients admitted to hospital via the ED, along with other factors such as neoplasm, age, and HAIs [13]. A recent Italian study in non-traumatic patients over 80 years confirmed that NEWS provided acceptable short-term prognostic accuracy for targeted risk stratification in this population [22]. In our series, NEWS was of moderate risk in 8.9% of cases and high-risk in 6.2% of cases, with higher percentages observed in patients with HABSIs compared to those without HABSIs (Figure 2). The high-risk score was confirmed as one of the four most important predictor variables selected by the logistic model.

In a significant number of patients, the cancer diagnosis is made after visiting the ED, and in these cases, the 12-month mortality resulted in higher rates than in cases in which the cancer diagnosis was obtained in other settings [23]. Advanced stages of cancer can increase susceptibility to HABSI due to neutropenia and impairment of the mucosal barrier; the use of central venous catheters is one of the main risk factors for HABSIs. This condition is exacerbated by the immunosuppression caused by cancer itself or recent treatments [24]. The types of cancer with the highest risk of infection and death are those of the pancreas, lung, biliary tract, anaplastic thyroid, and head and neck [25]. Our findings confirm the link between a cancer diagnosis and a poor prognosis, particularly for those of the pancreas, lung and colon. 

Like cancer, digestive system diseases can also cause HABSIs through a mechanism involving intestinal colonisation and systemic translocation by multidrug-resistant *Enterobacterales* [26]. In our dataset, patients with a primary diagnosis of gastrointestinal disease were found to be at risk of HABSI. Of the 2365 cases with diseases of the digestive system, the most common diagnoses at the time of admission were gallstones, cholecystitis and cholangitis (N = 336; 14.2%), intestinal obstructions (N = 274; 11.6%) and acute pancreatitis (N = 193; 8.2%).

High comorbidity burden is considered an important risk factor for HABSIs, indicating that underlying chronic conditions increase susceptibility to HABSI [27]. In our study, the CCI is confirmed as a predictive factor for patient risk stratification and the prediction of adverse outcomes, particularly in those classified as high-risk. The CCI resulted in high-risk (≥4) in 50.1% of cases, with a higher prevalence (62.1%) among deceased patients compared to survivors (50.0%). Diabetes, particularly in elderly individuals with poor glycaemic control, is confirmed as a major risk factor for infection [28]. Diabetes was found to be the most common comorbidity (17.3%), followed by chronic kidney disease (11.2%) and liver disease (11.2%). All these conditions were more prevalent in the HABSI group than in the non-HABSI group.

The onset of a HABSI is associated with an increased risk of complications and higher mortality in the short and medium term (30 days) [29]. HABSI 30-day mortality ranged from 13% to 26% in a French study [30] and from 20% to 28% in a Finnish study [31]. In our study, the 30-day mortality rates were 25.6% among patients who had developed a HABSI and 6.8% among those who had not. We believe that the mortality rate among our patients is among the highest compared with that of other case series, probably due to the characteristics of our patients, who were older, had a higher comorbidity index and presented with a more severe clinical profile.

This is the first study to identify specific risk factors for nosocomial infections in patients admitted to an acute care hospital’s ED with non-infectious primary diseases. However, the risk factors identified in published studies vary widely as they are heavily influenced by specific clinical contexts and patient populations. For example, septicaemia in the intensive care unit has been associated with previous antimicrobial therapy, the use of central venous catheters, and mechanical ventilation [32]. Conversely, acquired infections in general, ordinary wards have been linked to temperatures >38 °C (>100.4 °F), male sex and platelet counts <150,000/μL [33], whereas post-surgical pneumonia in patients undergoing valvular heart surgery correlates with elevated troponin and NT-proBNP values [34].

Although advanced age (particularly in the over-eighty age group) and female gender are considered variables potentially associated with the risk of HAI [35], they were not included in the stepwise procedure of the logistic model. Greater standardization of both research questions and the relevant variables used to predict outcomes will enable us to better understand which types of patients to study in the appropriate clinical setting for future research.

### 4.2. Limitations

Several limitations must be acknowledged. First, the data were collected from multicentre first-level EDs in the same healthcare area. These results were obtained from an area with a uniform healthcare organization; it may be that our results would not be confirmed if the level or organisation of health services differed from that in the study area.

Second, we used ICD9-CM diagnosis codes to identify HABSIs. Retrospective analysis of cases could generate a risk of selection bias, but the selection criteria we used should minimise the risk of false positives. All data were obtained from a database from four hospitals within a single Local Health Authority, which may limit external generalizability. Although ICD-9-CM codes are administrative by nature, each HABSI code was individually verified against the medical record by clinicians and administrative staff, and we confirmed correct diagnosis-code assignment. In addition, considering this internal validation, differences in regional health-care organization and coding practices could affect replication elsewhere.

Third, although we cannot be certain that our criteria for identifying cases without suspected infection at the time of admission are adequate, by excluding cases with a hospital stay of ≤3 days, cases diagnosed with an infectious disease, and those with an infectious disease or HABSI as their primary or secondary diagnosis, we believe we have at least significantly reduced the risk of including cases in whom the infection was already present. However, as this is a retrospective study, this risk must unfortunately be accepted and cannot be eliminated.

Fourth, the accuracy of the logistic model in predicting the event was 0.703 [SE 0.027], with an optimal cutoff point of 0.469, a level of discriminatory power considered only just acceptable for clinical observational studies. For example, other variables associated with the risk of HABSI could increase the model’s predictive power, such as indicators of disease severity, indicators of complications that have arisen, and procedures associated with a risk of infection. In our series, we considered procedures associated with a risk of infection, such as central venous catheters, urinary catheters, intubation, emergency pericardiocentesis, and pleurostomies, but they were so rare that it was not possible to include them among the variables to be tested in the logistic model. The only one represented was the urinary catheterisation, present in 18% of cases, but with no association with HABSI.

Unfortunately, it is not possible to understand whether the group of selected variables has such a high impact on the accuracy of the patient’s medium- and long-term prognosis. To achieve this result, it would be essential to minimise the risk of distortion through careful selection of the variables to be tested, good outcome measures, and careful analysis of the results, eliminating the so-called self-fulfilling prophecy effect. Additional clinical events, such as complications and severity scores, with appropriate use of statistical models (e.g., survival analysis including time to event or Bayesian models), could improve predictive performance.

Fifth, an approach based on the time of event onset, which could take into account competing risks such as death, would be the ideal framework for modelling the onset of HABSIs. However, the retrospective database used for this study did not contain reliable temporal data regarding the onset of a HABSI, but only information regarding the occurrence of the event during hospitalization. Consequently, since it was not possible to determine the exact time of event onset required for survival or competing risk analyses, we used a multivariate analysis that did not account for the time of the event.

Sixth, we acknowledge that among subjects diagnosed as non-infectious, we included a group of diagnoses, particularly those involving the digestive system, which, by definition, could be caused by infections (e.g., cholangitis, cholecystitis, colonic diverticulitis, and acute pancreatitis). This finding could be a limitation; however, also considering the risk, it would not be possible to determine retrospectively whether these cases were of infectious origin or not.

### 4.3. Generalizability

Although the sample analysed was large, the data were collected from four first-level EDs in a homogeneous healthcare area. Therefore, we cannot rule out the possibility that the results may not be fully replicable in areas with different organisational models.

## 5. Conclusions

A set of clinical features identified during an ED visit can be associated with the onset of a HABSI with a just acceptable degree of accuracy, even if the patients did not present with any suspected symptoms of an infectious condition at the time of the evaluation in the ED. Early identification of the profile of these patients could prevent complications and improve prognosis. Prospective multicentre studies could improve these findings.

## Figures and Tables

**Table 1:** Baseline characteristics of the two groups in relation to the presenting clinical profile.

	Total, No.	HABSI, No. (%)	Non HABSI, No. (%)	OR (95% CI)	*p*-Value
Patients	18,304	414 (92.3)	17,890 (97.7)	n.c.	n.c.
**Sex** (males)	9034 (49.9)	235 (56.8)	8799 (49.2)	1.36 (1.11–1.65)	0.002
**Age** (years)	75 (16)	77 (14)	76 (16)	n.c.	n.c.
18–30	505 (2.8)	6 (1.4)	499 (2.8)	0.51 (0.23–1.15)	0.100
31–40	432 (2.4)	3 (0.7)	429 (2.4)	0.30 (0.10–0.93)	0.027
41–50	770 (4.2)	11 (2.7)	759 (4.2)	0.62 (0.34–1.13)	0.112
51–60	1470 (8.0)	28 (6.8)	1442 (8.1)	0.83 (0.56–1.22)	0.337
61–70	2363 (12.9)	56 (13.5)	2307 (12.9)	1.06 (0.79–1.41)	0.705
71–80	4343 (23.7)	101 (24.4)	4242 (23.7)	1.04 (0.83–1.30)	0.746
>80	8421 (46.0)	209 (50.6)	8212 (45.9)	1.20 (0.99–1.46)	0.065
**NEWS**					
0–3	15,544 (84.9)	306 (73.9)	15,238 (85.2)	0.49 (0.39–0.62)	<0.001
4–6	1623 (8.9)	50 (12.1)	1573 (8.8)	1.42 (1.06–1.92)	0.020
>6	1137 (6.2)	58 (14.0)	1079 (6.0)	2.54 (1.91–3.37)	<0.001
**CCI**					
1–2	3333 (18.2)	39 (9.4)	3294 (18.4)	0.46 (0.33–0.64)	<0.001
3–4	5771 (31.5)	118 (28.5)	5653 (31.6)	0.86 (0.70–1.07)	0.180
>4	9200 (50.3)	257 (62.1)	8943 (50.0)	1.64 (1.34–2.0)	<0.001

Data are presented as number of cases (N) and percentages (%), with odds ratios (OR) and 95% confidence intervals (95% CIs). Statistical significance was set at a *p*-value of <0.001. NEWS: New Early Warning Score and CCI: Charlson Comorbidity Index, (n.c.) not calculable.

**Table 2:** Patient characteristics concerning ICD-9-CM diagnosis codes, listed in order of importance, reported as the number of cases (%), with differences between HABSI and non-HABSI groups as means with 95% confidence intervals (95% CIs). A *p*-value < 0.05 was considered statistically significant. ( - ): Indicates that the value is zero or undetectable.

ICD-9 Diagnosis Codes	Total No. (%)	HABSI No. (%)	Non-HABSI No. (%)	Difference (95% CI)	*p*-Value
Diseases of the Respiratory System (460–519)	4749 (26.0)	116 (28.0)	4633 (25.9)	1.11 (0.90, 1.38)	0.330
Diseases of the Circulatory System (390–459)	4077 (22.3)	68 (16.4)	4009 (22.4)	0.68 (0.52, 0.88)	0.004
Diseases of the Digestive System (520–579)	2365 (12.9)	72 (17.4)	2293 (12.8)	1.43 (1.11–1.85)	0.006
Injury and Poisoning (800–999)	2095 (11.4)	18 (4.3)	3300 (14.0)	0.35 (0.22–0.56)	<0.001
Diseases of the Genitourinary System (580–629)	1623 (7.7)	51 (12.3)	1572 (8.8)	1.46 (1.08–1.96)	0.012
Neoplasms (140–239)	1021 (5.6)	43 (10.4)	978 (5.5)	2.0 (1.45–2.77)	<0.001
Symptoms, Signs & Laboratory Findings (780–799)	514 (2.8)	9 (2.2)	505 (2.8)	0.77 (0.39–1.49)	0.429
Diseases of the Blood (280–289)	410 (2.2)	8 (1.9)	402 (2.2)	0.86 0.42–1.74)	0.669
Mental Disorders (290–319)	403 (2.2)	2 (0.5)	401 (2.2)	0.21 (0.05–0.85)	0.016
Endocrine, Nutritional, and Metabolic (240–279)	274 (1.5)	7 (1.7)	267 (1.5)	1.14 (0.53–2.42)	0.742
Diseases of the Nervous System (320–389)	379 (2.0)	7(1.7)	372 (2.1)	0.81 (0.38–1.72)	0.583
Diseases of the Musculoskeletal System (710–739)	189 (1.0)	9 (2.2)	180 (1.0)	2.19 (1.11–4.30)	0.020
Diseases of the Skin (680–709)	94 (0.5)	3 (0.7)	91 (0.5)	1.43 (0.45–4.53)	0.543
Complications of Pregnancy (630–679)	58 (0.3)	0 (0.0)	58 (0.3)	--	–
External Causes (E, V codes)	39 (0.2)	1 (0.2)	38 (0.2)	12.14 (0.16–8.30)	0.899
Congenital Malformations (740–759)	14 (0.1)	0 (0.0)	14 (0.1)	--	--

## Data Availability

The current study’s generated/analysed datasets are available upon request.

## Abbreviations

The following abbreviations are used in this manuscript:

CCI	Charlson Comorbidity Index
ECDC	European Centre for Disease Prevention and Control
ED	emergency department
EDLoS	emergency department length of stay
HABSI	healthcare-associated bloodstream infection
HAI	healthcare-associated infection
NEWS	National Early Warning Score
ROC	Receiver Operating Characteristic

## References

[1] Markwart R., Saito H., Harder T., Tomczyk S., Cassini A., Fleischmann-Struzek C., Reichert F., Eckmanns T., Allegranzi B. (2020). Epidemiology and Burden of Sepsis Acquired in Hospitals and Intensive Care Units: A Systematic Review and Meta-Analysis. Intensive Care Med..

[2] Point Prevalence Survey of Healthcare-Associated Infections and Antimicrobial Use in European Acute Care Hospitals—2022-2023. https://www.ecdc.europa.eu/en/publications-data/PPS-HAI-AMR-acute-care-europe-2022-2023.

[3] Prowle J.R., Echeverri J.E., Ligabo E.V., Sherry N., Taori G.C., Crozier T.M., Hart G.K., Korman T.M., Mayall B.C., Johnson P.D. (2011). Acquired Bloodstream Infection in the Intensive Care Unit: Incidence and Attributable Mortality. Crit. Care.

[4] Lamy B., Dargère S., Arendrup M.C., Parienti J.-J., Tattevin P. (2016). How to Optimize the Use of Blood Cultures for the Diagnosis of Bloodstream Infections? A State-of-the Art. Front. Microbiol..

[5] Friedman N.D., Kaye K.S., Stout J.E., McGarry S.A., Trivette S.L., Briggs J.P., Lamm W., Clark C., MacFarquhar J., Walton A.L. (2002). Health Care—Associated Bloodstream Infections in Adults: A Reason to Change the Accepted Definition of Community-Acquired Infections. Ann. Intern. Med..

[6] Liang S.Y., Theodoro D.L., Schuur J.D., Marschall J. (2014). Infection Prevention in the Emergency Department. Ann. Emerg. Med..

[7] Phungoen P., Lerdprawat N., Sawanyawisuth K., Chotmongkol V., Ienghong K., Sumritrin S., Apiratwarakul K. (2021). Clinical Factors Associated with Bloodstream Infection at the Emergency Department. BMC Emerg. Med..

[8] Chase M., Klasco R.S., Joyce N.R., Donnino M.W., Wolfe R.E., Shapiro N.I. (2012). Predictors of Bacteremia in Emergency Department Patients with Suspected Infection. Am. J. Emerg. Med..

[9] World Medical Association (2013). World Medical Association Declaration of Helsinki: Ethical Principles for Medical Research Involving Human Subjects. JAMA.

[10] Moons K.G.M., Altman D.G., Reitsma J.B., Ioannidis J.P.A., Macaskill P., Steyerberg E.W., Vickers A.J., Ransohoff D.F., Collins G.S. (2015). Transparent Reporting of a Multivariable Prediction Model for Individual Prognosis or Diagnosis (TRIPOD): Explanation and Elaboration. Ann. Intern. Med..

[11] Grafstein E., Unger B., Bullard M., Innes G. (2003). Canadian Emergency Department Information System (CEDIS) Presenting Complaint List (Version 1.0). CJEM.

[12] Charlson M.E., Pompei P., Ales K.L., MacKenzie C.R. (1987). A New Method of Classifying Prognostic Comorbidity in Longitudinal Studies: Development and Validation. J. Chronic Dis..

[13] Raoofi S., Kan F.P., Rafiei S., Hosseinipalangi Z., Mejareh Z.N., Khani S., Abdollahi B., Talab F.S., Sanaei M., Zarabi F. (2023). Global Prevalence of Nosocomial Infection: A Systematic Review and Meta-Analysis. PLoS ONE.

[14] Agresti A., Caffo B. (2000). Simple and Effective Confidence Intervals for Proportions and Differences of Proportions Result from Adding Two Successes and Two Failures. Am. Stat..

[15] Williams B. (2022). The National Early Warning Score: From Concept to NHS Implementation. Clin. Med. Lond. Engl..

[16] Fluss R., Faraggi D., Reiser B. (2005). Estimation of the Youden Index and Its Associated Cutoff Point. Biom. J..

[17] Ali H., Salleh M.N.M., Hussain K., Ahmad A., Ullah A., Muhammad A., Naseem R., Khan M. (2019). A Review on Data Preprocessing Methods for Class Imbalance Problem. Int. J. Eng..

[18] Boehme A.K., Kumar A.D., Dorsey A.M., Siegler J.E., Aswani M.S., Lyerly M.J., Monlezun D.J., George A.J., Albright K.C., Beasley T.M. (2013). Infections Present on Admission Compared with Hospital-Acquired Infections in Acute Ischemic Stroke Patients. J. Stroke Cerebrovasc. Dis. Off. J. Natl. Stroke Assoc..

[19] Higgins T.L., Deshpande A., Zilberberg M.D., Lindenauer P.K., Imrey P.B., Yu P.-C., Haessler S.D., Richter S.S., Rothberg M.B. (2020). Assessment of the Accuracy of Using ICD-9 Diagnosis Codes to Identify Pneumonia Etiology in Patients Hospitalized With Pneumonia. JAMA Netw. Open.

[20] Kärki T., Plachouras D., Cassini A., Suetens C. (2019). Burden of Healthcare-Associated Infections in European Acute Care Hospitals. Wien. Med. Wochenschr..

[21] Gao Y., Xiang L., Yi H., Song J., Sun D., Xu B., Zhang G., Wu I.X. (2025). Confounder Adjustment in Observational Studies Investigating Multiple Risk Factors: A Methodological Study. BMC Med..

[22] Covino M., Cacciamani Fanelli P.M., Bonadia N., Maccauro V., Della Polla D.A., De Matteis G., Piccioni A., Gasbarrini A., Sandroni C., Franceschi F. (2026). Early Warning Scores in Emergency Department Patients Aged 80 Years or Older. JAMA Netw. Open.

[23] Pettit N., Sarmiento E., Kline J. (2022). Disparities in Outcomes among Patients Diagnosed with Cancer in Proximity to an Emergency Department Visit. Sci. Rep..

[24] Magill S.S., O’Leary E., Janelle S.J., Thompson D.L., Dumyati G., Nadle J., Wilson L.E., Kainer M.A., Lynfield R., Greissman S. (2018). Changes in Prevalence of Health Care-Associated Infections in U.S. Hospitals. N. Engl. J. Med..

[25] MacPhail A., Dendle C., Slavin M., McQuilten Z. (2024). Hospital-Acquired Bloodstream Infections in Patients with Cancer: Current Knowledge and Future Directions. J. Hosp. Infect..

[26] Henoun Loukili N., Perrin A., Gaillot O., Bruandet A., Boudis F., Sendid B., Nseir S., Zahar J.-R. (2025). Is Intestinal Colonization with Multidrug-Resistant Enterobacterales Associated with Higher Rates of Nosocomial Enterobacterales Bloodstream Infections?. Int. J. Infect. Dis. IJID Off. Publ. Int. Soc. Infect. Dis..

[27] Cantero M., Salamanca C., Parra L.M., González-Pérez M.E., García de la Vega M., Asensio Á. (2026). Attributable Mortality to Healthcare-Associated Infections: A Comprehensive Nationwide Assessment in Spain, 2022 and 2023. Eurosurveillance.

[28] Holt R.I.G., Cockram C.S., Ma R.C.W., Luk A.O.Y. (2024). Diabetes and Infection: Review of the Epidemiology, Mechanisms and Principles of Treatment. Diabetologia.

[29] Buetti N., Tabah A., Setti N., Ruckly S., Barbier F., Akova M., Aslan A.T., Leone M., Bassetti M., Morris A.C. (2024). The Role of Centre and Country Factors on Process and Outcome Indicators in Critically Ill Patients with Hospital-Acquired Bloodstream Infections. Intensive Care Med..

[30] Pepin C.S., Thom K.A., Sorkin J.D., Leekha S., Masnick M., Preas M.A., Pineles L., Harris A.D. (2015). Risk Factors for Central Line-Associated Bloodstream Infections: A Focus on Comorbid Conditions. Infect. Control Hosp. Epidemiol..

[31] Kontula K.S., Skogberg K., Ollgren J., Järvinen A., Lyytikäinen O. (2022). Early Deaths Associated with Community-Acquired and Healthcare-Associated Bloodstream Infections: A Population-Based Study, Finland, 2004 to 2018. Eurosurveillance.

[32] Magrini E., Rando E., Liguoro B., Salvati F., Vecchio P.D., Fantoni M., Torti C., Murri R. (2025). Risk Factors Associated with Bloodstream Infections Caused by *Acinetobacter baumannii* in Hospital Settings: A Systematic Review and Meta-Analysis. CMI Commun..

[33] Singh H., Sheth R., Bhatia M., Muhammad A., Bachour C., Metcalf D., Kak V. (2024). Clinical Predictors of Hospital-Acquired Bloodstream Infections: A Healthcare System Analysis. Spartan Med. Res. J..

[34] Duchnowski P., Śmigielski W. (2023). Risk Factors of Postoperative Hospital-Acquired Pneumonia in Patients Undergoing Cardiac Surgery. Medicina.

[35] Ding M., Ning Y., Song L., Yu X., Yan B., Li P., Tian W., Zhang R., Chen W., Zhen J. (2025). Clinical Features and Risk Factors of Older Adults with Bloodstream Infection. BMC Geriatr..

